# Justification trajectories for pension inequality in Chile (2016–2023): the role of social class and beliefs in meritocracy

**DOI:** 10.3389/fsoc.2026.1771856

**Published:** 2026-03-10

**Authors:** Juan Carlos Castillo, René Canales-Sellés, Andreas Laffert, Tomás Urzúa

**Affiliations:** 1 Department of Sociology, University of Chile, Santiago, Chile; 2 Instituto de Sociología, Pontificia Universidad Católica de Chile, Santiago, Chile

**Keywords:** Chile, pension system, market justice, meritocracy, social class

## Abstract

**Introduction:**

The Chilean pension system, characterized by full privatization and individual capitalization, plays a central role in shaping old-age inequality. Despite widespread social unrest and demands for reform, a segment of the population continues to justify income-based pension differences, a phenomenon conceptualized as preferences for pension market justice. This study investigates the interplay between social class, meritocratic beliefs (distinguishing between effort and talent), and preferences for market justice in the Chilean pension system between 2016 and 2023.

**Methods:**

Using six waves of panel data from the Chilean Longitudinal Social Survey-ELSOC (*N*_*observations*_ = 5,755; *N*_*individuals*_ = 1,027), the analysis employs Cumulative Link Mixed Models (CLMM) to examine longitudinal changes. This approach allows for the decomposition of variance into between-person and within-person effects, assessing how stable class positions and evolving meritocratic perceptions influence the legitimacy of market-based pension allocation.

**Results:**

The findings reveal a “class neutralization” phenomenon, where objective social class does not significantly predict support for pension market justice. Instead, support has grown across all groups. Meritocratic perceptions of effort are consistently associated with higher market justice preferences at both between- and within-person levels. Conversely, talent-based perceptions do not show a main effect but interact significantly with Specific Class Positions: they predict higher support among the intermediate class (between-person) and among unemployed individuals whose perceptions of talent rewards increase over time (within-person).

**Discussion:**

These findings suggest that the institutional architecture of the pension system frames outcomes as results of individual responsibility, dampening class-based redistributive demands. Effort-based meritocracy acts as a key legitimizing ideology for pension inequality. However, talent-based justifications appear to function differently, serving as a compensatory narrative for the intermediate class and the unemployed to rationalize distributive outcomes in a context where the link between effort and reward is structurally constrained.

## 1 Introduction

The pension system lies at the core of the welfare state, shaping risk exposure among the elderly population (Breznau, 2023; Esping-Andersen, 1990). Such a system is based on the common understanding that the elderly require financial support after retirement, which is accumulated during working life with varying degrees of intergenerational solidarity (OECD, 2023). In this understanding, the principles of justice and redistributive beliefs are crucial, as they shape how individuals perceive the fairness of pension systems and the distribution of benefits (Liebig and Sauer, 2016). For instance, in systems where solidarity is prominent, there may be greater support for redistributive mechanisms to ensure a more equitable distribution of pension benefits across different social groups. Conversely, in societies where meritocratic beliefs are prevalent, individuals may justify inequalities in pension benefits on the grounds of factors such as work history, contributions to the system, and individual effort (Castillo et al., 2024; Mau, 2015; Mijs, 2021).

The Chilean pension system provides a unique setting for examining the principles of justice governing pension benefits, given its extensive experience with pension privatization. In 1981, Chile became the first country to replace a public pay-as-you-go scheme with a fully privatized, defined-contribution system based on mandatory individual capitalization accounts administered by private financial institutions (Solimano, 2021). Under this model, individuals with higher earnings and stable contribution histories receive substantially higher pensions, whereas those with lower and more irregular incomes, especially workers in low-prestige occupations and women, receive the opposite (Arenas, 2019; Madero-Cabib et al., 2019). This system has generated marked inequalities and heightened poverty risks in old age, leaving many struggling to make ends meet (Borzutzky and Hyde, 2016; Cabib, 2025; Gálvez et al., 2024; Subsecretaría de Previsión Social., 2023). In this context, widespread public discontent and powerful social movements demanding far-reaching pension reforms have emerged in recent years (Somma et al., 2021). At the same time, recent evidence shows that an essential and growing share of the population continues to justify income-based differences in pension benefits on the grounds of individual contributions—that is, to endorse market-based access to pensions (Castillo et al., 2025). Regarding this, understanding how meritocratic beliefs and social class shape preferences for market-based pension benefits is crucial for informing policy debates and advancing social justice (Busemeyer and Iversen, 2020; Svallfors, 2007).

Despite the uniqueness of the Chilean pension system and its discontents, to our knowledge, there are no studies that have analyzed popular support for this system, let alone from a longitudinal perspective. Filling this gap, the present research examines the evolution of market justice preferences in the area of pensions during the last decade Chile. The concept of market justice refers to the belief that the distribution of goods and services (as pensions) should be determined by mechanisms such as supply and demand, rather than by social or political considerations (Lane, 1986; Lindh, 2015). Research on market justice beliefs focuses on the extent to which societies support the idea that payment capacity and individual contribution are the primary criteria for resource allocation, emphasizing personal responsibility and effort over collective welfare (Castillo et al., 2024; Kluegel et al., 1999). The concept of market justice, although related to redistributive preferences, covers a different dimension, as it focuses on the justification of inequalities based on market mechanisms rather than on the desire to reduce or increase such inequalities (Alesina and Angeletos, 2005; Alesina and La Ferrara, 2005).

In this study, we argue that market justice preferences in pensions are closely linked to both class belonging and meritocratic beliefs. On the one hand, social class has been shown to influence individuals' perceptions of justice and fairness in various social contexts, whereby individuals from higher social classes may be more likely to justify market-based pensions based on their rational interests (Busemeyer and Iversen, 2020; Svallfors, 2007). On the other hand, meritocracy is a widely held belief that individuals should be rewarded based on their abilities and efforts (McNamee and Miller, 2004; Young, 1962), which could lead to justifying larger inequalities in pension benefits based on individual contributions. To address these issues, we use representative panel data from the Chilean Longitudinal Social Survey (ELSOC), a national six-wave panel study fielded between 2016 and 2023 to examine attitudes, emotions, and behaviors among adults aged 18 to 75 in relation to social cohesion and conflict. The analytical sample yielded a two-level data set of *N* = 5,755 observations nested within *N* = 1,027 individuals.

## 2 Theoretical and empirical background

### 2.1 Market justice preferences

In recent years, welfare states have undergone several institutional transformations, with market expansion strongly penetrating the field of social services in parallel to public provision (Busemeyer and Iversen, 2020). Such processes have led to changes in the architecture of the institutions responsible for social welfare. On the one hand, private companies operate as service administrators of benefits based on the user's ability to pay, whereas on the other hand, public providers have reorganized to compete as quasi-markets (Lindh, 2017). Besides changes at the formal level, the commodification of social services shapes cultural norms and beliefs, an institutional impact referred to as *policy feedback effect* (Baland and Schlager, 2019; Jaime-Castillo, 2013). In this sense, markets operate as moral projects (Fourcade and Healy, 2007).

In the field of distributive justice, market preferences have gained relevance in attitudinal studies on redistribution. A seminal study in this area was performed by Kluegel and Smith (1981), analyzing the normative bases of beliefs about social stratification. In this context, market justice preferences emerge as a concept related to a normative principle that legitimizes access to goods and services based on an individual's or family's ability to pay (Lane, 1986). This market-oriented redistribution is closely related to the justification of inequalities, as it views the market as a space of equal opportunity, where economic success is understood as an individual outcome (Kluegel et al., 1999; Kluegel and Smith, 1981). In this way, market justice legitimizes socioeconomic inequalities from an economic and moral perspective, ignoring the structural conditions that generate these disparities.

Within the market justice framework, pensions are one of the central elements due to their centrality in discussions of economic distribution among the elderly population (Arenas, 2019). Some pension systems are based on principles of solidarity, whereas others are based on privatization policies that manage resources according to a market logic (Arrizabalo Montoro et al., 2019). Such varying approaches certainly have cultural implications for societies. For example, market-oriented plans are associated with feelings of insecurity due to the individualization of risk (Yen, 2018). Therefore, distributive principles that shape pension systems could be mirrored in market justice preferences (Castillo et al., 2025).

Recent literature on market preferences has found relevant associations with both individual and contextual factors. Various studies suggest that individuals with higher socioeconomic status tend to exhibit greater market preferences than those in more disadvantaged social positions (Koos and Sachweh, 2019; Lindh, 2015). Similarly, political ideology has been linked to preferences for market justice, with right-wing individuals mostly supporting market-oriented distribution (Castillo et al., 2024; Lee and Stacey, 2024). These trends reflect a moral economy based on the justification of status by those who benefit from the market. Regarding contextual factors, the evidence suggests that in countries with greater private investment in services, individuals tend to decrease their preferences for redistribution, internalizing the market order as a criterion of justice, whereas the opposite occurs in countries where public provision of services prevails (Busemeyer, 2013; Immergut and Schneider, 2020; Lindh, 2015).

Most of the research in this area have relied on survey data. In such studies, questions about market justice ask directly about the support for the provision of social services (as education, pensions, and health) based on individual payment capacity (i.e.: “Is it fair that high-income individuals have better pensions than lower-income individuals?”) (Busemeyer, 2013; Kerner, 2020; Lindh, 2015). The International Social Survey Programme (ISSP) has been a key resource for developing a market justice agenda. Based on the ISSP measurement proposal, some researches have measured market preferences using an index constructed from the corresponding items (Castillo et al., 2025), whereas others analyze the items separately. Nevertheless, market justice preferences in pensions are a sidelined area in this agenda, as ISSP does not include an item referring to pensions (only health and education).

### 2.2 Meritocracy

Meritocracy is a normative principle that justifies the distribution of goods and services based on individual effort and talent, emphasizing that rewards should correspond to personal achievements rather than social status or inherited privileges (Sandel, 2020; Young, 1962). This concept has gained prominence in contemporary societies, where meritocratic ideals are often invoked to legitimize socioeconomic inequalities, suggesting that individuals' success or failure is primarily determined by their own merits (Littler, 2018).

Research on meritocratic beliefs has explored how individuals perceive the fairness of social systems that prioritize merit-based distribution, linked to individual and contextual factors. Studies have shown that people who strongly endorse meritocratic ideals tend to justify existing inequalities, attributing success to personal effort and downplaying the role of structural factors such as social class (Reynolds and Xian, 2014). This perspective can lead to reduced support for redistributive policies, as individuals may view such interventions as undermining the principles of meritocracy (Piketty, 2014). Besides, individuals with higher socioeconomic status are more likely to endorse meritocratic ideals, as they often benefit from systems that reward their efforts and talents (Harrs and Sterba, 2025). Additionally, political ideology plays a significant role, with right-leaning individuals typically exhibiting stronger support for meritocracy compared to their left-leaning counterparts. Regarding contextual factors, the evidence is twofold and even contradictory. Although in societies with high levels of social mobility and equal opportunities individuals may be more inclined to endorse meritocratic ideals, some evidence suggests that inequality is linked to larger meritocratic perception, a phenomenon known as the *paradox of meritocracy* (Mijs, 2021). Overall, the concept of meritocracy remains a central theme in discussions about social justice and inequality, highlighting the complex interplay between individual effort, structural conditions, and societal values.

The measurement of meritocratic beliefs has been approached through various survey instruments that assess individuals' attitudes toward effort, talent, and the fairness of merit-based systems. Commonly used items include questions about the importance of hard work for success, the role of innate ability, and perceptions of fairness in competitive environments (Castillo et al., 2023). In the extant literature, the measurement of meritocratic beliefs has often been inconsistent, with different studies employing varying scales and items, making cross-study comparisons challenging. To address this issue, some researchers have advocated for the development of standardized measures that capture the multifaceted nature of meritocratic beliefs. A first distiction to make here is the one between perceptions and preferences for meritocracy, usually the first being descriptive and the second more normative in nature (Castillo et al., 2023). Research on meritocratic perceptions have revealed that people's beliefs about the extent to which meritocracy exists in society can differ significantly from their normative support for meritocratic principles (Castillo et al., 2023). Besides, whereas meritocratic preferences are usually more consensual, meritocratic perceptions tend to vary more across individuals and contexts. In this line, we argue that meritocratic perceptions, this is, that effort and talent are viewed as mechanisms to get ahead in society, are positively associated with support for market justice preferences.

### 2.3 Market justice and social class

Despite longstanding critiques of class analysis (Pakulski and Waters, 1997), research continues to show that class belonging remains central for understanding attitudes toward inequality (Dodson, 2017; Kulin and Svallfors, 2013; Lindh, 2015; Lindh and McCall, 2020). Across different class approaches, a shared premise is that socioeconomic positions organize resources, risks, and life chances, thereby shaping both material interests and shared experiences that inform how people interpret social reality and evaluate the fairness of unequal outcomes (Bottero, 2004; Langsåther and Evans, 2020; Svallfors, 2006). In this context, class is analytically relevant as it links objective stratification to subjective evaluations of justice.

In welfare-state theory, labor market positions define similarities in economic resources, opportunities, and constraints, structuring both economic interests and group identities (Erikson and Goldthorpe, 1992a; Korpi and Palme, 2003). When distribution is primarily mediated by markets, access and outcomes are more closely tied to earnings capacity and exposure to labor market risks. In contrast, political distribution based on social citizenship can buffer market dependence, particularly for groups with fewer marketable resources (Korpi, 2006, 2018). A central implication is that individuals in advantaged class positions—who rely less on social protections and can convert earnings into welfare—should, on average, be more willing to accept market-based allocation than those in disadvantaged positions. Privileged classes, characterized by higher and more stable earnings and formal employment, are more likely to sustain continuous contributions and to accumulate larger balances, resulting in higher pensions (Gil-Hernández et al., 2025). Conversely, working-class trajectories more often involve unemployment, informality, and lower wages, producing fragmented contribution histories and lower savings, which reproduce and amplify labor-market inequalities into old age (Arrizabalo Montoro et al., 2019; Cabib, 2025). Under these conditions, an interest-based expectation is that upper-class respondents will exhibit higher average support for market-based pension inequalities than lower-class respondents.

Comparative evidence supports the expectation of interest-based market justice: higher-income, better-educated, and higher-class individuals are more likely to endorse market-based distributive principles (Busemeyer, 2015; Immergut and Schneider, 2020; Lindh, 2015; Svallfors, 2007). In the pension domain, institutional design can reinforce class-based incentives: where private provision is available, higher-income groups have greater scope to rely on market-based arrangements, which may reduce their attachment to public pension spending (Busemeyer and Iversen, 2020). Consistent with this view, some evidence from Latin America and Chile links material advantage and positive evaluations of pensions, showing higher support for market allocation among higher-income and university-educated respondents across welfare domains (Castillo et al., 2024; Kerner, 2020; Otero and Mendoza, 2024).

Class, however, should not only structure average levels of pension market justice; it should also condition how meritocratic beliefs translate into these preferences, both between individuals and within individuals over time. Meritocracy provides a normative rationale for unequal outcomes by framing them as deserved returns to effort and talent, yet its plausibility is not uniform across the class structure. Because upper-class individuals more often benefit from the earnings–contributions–benefits chain, meritocratic interpretations are likely to be more congruent with their lived outcomes and interests (Castillo et al., 2025; Lindh and McCall, 2020). Consequently, meritocratic beliefs should be more strongly associated with support for pension market justice among advantaged classes than among disadvantaged classes. For the same reason, when individuals revise their meritocratic perceptions over time, these within-person changes should reinforce support for market-based welfare more strongly among upper-class respondents—where they resonate with institutionalized signals of “performance” in individually funded systems (e.g., balances and returns)—than among lower-class respondents, for whom the alignment between merit-based reward narratives and typical pension prospects is weaker (Kerner, 2020).

### 2.4 The Chilean context

Despite sustained economic growth, Chile remains one of the most unequal countries in Latin America and the OECD. The poorest 50% of the population earns only 8.2% of total income and holds around 3% of net wealth, while the richest 1% receive almost 27% of all income and control 37% of the country's wealth (Chancel et al., 2025), a pattern that has changed little since the 1990s (Flores et al., 2020). These inequalities are not simply the outcome of market forces; they are rooted in neoliberal reforms that privatized and commodified key spheres of social reproduction (Ferre, 2023; PNUD, 2017). Introduced under the military dictatorship (1973–1989) and subsequently expanded by democratic governments, these reforms entrenched market logics in essential public services through concessions and regulatory frameworks that favored private provision, producing a model of commodified public services that underpins Chile's neoliberal order (Ffrench-Davis, 2018; Madariaga, 2020). Health and education have been extensively studied in this regard (Llambías-Wolff, 2019; Rosenzvaig-Hernandez, 2024), revealing structurally segmented systems in which access and quality depend heavily on the ability to pay (Castillo et al., 2025). Pensions represent a paradigmatic case of this broader welfare marketization, unfolding under conditions of high inequality and market-based provision in a core domain of social protection.

Since 1981, old-age income security in Chile has been organized primarily through a fully privatized, mandatory defined-contribution scheme. A far-reaching reform replaced the previous earnings-related, pay-as-you-go social insurance system with individual capitalization accounts administered by private Pension Fund Administrators (AFP) (Huber and Stephens, 2000; Solimano, 2021). Under this design, dependent employees must contribute 10% of their wages, plus an administrative fee, to individual accounts, while employers face no mandatory contributions. AFPs invest these savings in domestic and international financial markets. Since the early 2000s, affiliates have been assigned to, or have chosen among, several “multifunds” (A–E) with distinct risk–return profiles (Barriga and Kremerman, 2024; Hyde and Borzutzky, 2015). After more than four decades, benefit levels remain modest: for most retirees, AFP-financed pensions fall below the statutory minimum wage, and self-funded replacement rates hover around 30%, even among workers with long and dense contribution records (Gálvez et al., 2024; Madero-Cabib et al., 2019). In response, the state has progressively expanded tax-financed solidarity components—most notably the *Nuevo Pilar Solidario* (2008) and the *Pension Garantizada Universal* (PGU, 2022)—to top up low or non-existent contributory pensions among poorer and lower-middle older adults (Boccardo, 2020). This has produced a hybrid arrangement in which privately managed individual accounts are substantially subsidized by the state, and public transfers constitute a sizeable share of total pension income at the bottom of the distribution (Gálvez et al., 2024). This model not only shapes pension outcomes; it also frames pensions as the result of individual contributions and financial returns, making inequalities appear, at least in part, as earned and therefore potentially justifiable.

This institutional configuration both reflects and reinforces Chile's broader pattern of persistent inequality combined with extensive welfare privatization. Stratified access to social protection has eroded expectations of upward mobility and contributed to social unrest, with pensions becoming a focal point of public discontent and protest (PNUD, 2017; Somma et al., 2021). At the same time, the attitudinal landscape is not univocal. Alongside widespread criticism of the private pension system and repeated demands for reform, recent evidence suggests that a growing share of the population considers pension inequalities based on individual contribution histories to be fair or legitimate (Castillo et al., 2025). This coexistence points directly to the problem of pension market justice: how, and for whom, market-based principles of allocation become morally acceptable in the context of old-age security. These developments are embedded in broader transformations of Chile's class structure and moral orientations. Meritocratic beliefs have intensified, accompanying the diffusion of market-centered moral frameworks in which individuals and families are expected to act as self-responsible agents, investing in their own “market value” and bearing the risks of their life-course trajectories (Canales Cerón et al., 2021; Mau, 2015). In a context marked by high and durable inequalities, deep marketization of welfare, and the entrenchment of meritocratic beliefs, it becomes crucial to examine support for pension market justice, how it varies across social classes and meritocratic orientations, and how it has evolved (Castillo et al., 2025, 2024).

### 2.5 This study

Building on class-based accounts of distributive preferences, we expect support for pension market justice to vary systematically across the class structure. Prior research shows that higher class position is associated with lower support for redistributive policies and a stronger inclination toward market-based allocation (Koos and Sachweh, 2019; Lindh, 2015). In contexts where access and quality are more closely tied to purchasing power, advantaged groups face fewer constraints and risks, making market allocation both more feasible and more defensible as a distributive principle.

We further argue that meritocratic perceptions provide a key normative lens through which individuals evaluate pension inequalities. Existing work suggests that perceiving society as meritocratic is positively related to market-oriented preferences in welfare domains, including pensions, both cross-sectionally and over time (Castillo et al., 2025; Castillo et al., 2019). Individuals who see effort and talent as decisive for success are more likely to view income-linked differences as legitimate outcomes of individual contribution and deservingness (Castillo et al., 2024). Hence, meritocratic perceptions should be associated with stronger pension market justice preferences, not only between individuals but also as people revise these beliefs over time.

Finally, we examine whether class conditions the attitudinal implications of meritocracy. Because the earnings–contributions–benefits link is typically more favorable for advantaged groups, meritocratic narratives may be more congruent with their material position and experiences. We therefore expect the association between meritocracy and pension market justice preferences to be stronger among upper-class respondents. In addition, we explore whether within-person increases in meritocratic perceptions translate into larger changes in pension market justice among upper-class individuals than among those in lower classes.

Based on these arguments, we propose the following hypotheses:

*H*1;(Between-person): Those belonging to the upper classes show, on average, higher pension market justice preferences than those belonging to the lower classes.

*H*2_*a*_; (Between-person): Individuals with stronger meritocratic perceptions show, on average, higher pension market justice preferences.

*H*2_*b*_; (Within-person): When the same individual strengthens their meritocratic perceptions over time, their pension market justice preferences increase.

*H*3_*a*_; (Between-person): The positive association between meritocracy (BE) and pension market justice preferences is stronger in upper classes.

*H*3_*b*_; (Within-person): When upper-class individuals increase their meritocratic perceptions over time (WE), their pension market justice preferences rise more than in lower classes.

## 3 Data, variables, and methods

### 3.1 Data

This research draws on the Chilean Longitudinal Social Survey (ELSOC), a national panel study fielded between 2016 and 2023 to examine attitudes, emotions, and behaviors among adults aged 18 to 75 in relation to social cohesion and conflict. ELSOC uses a probabilistic, stratified, clustered, multistage sampling design covering urban centers and small towns. The sampling frame is proportionally stratified into six categories of urban population size; within these strata, households were selected through multistage procedures based on 1,067 sampled blocks.

ELSOC has been administered annually since 2016, except in 2020 when fieldwork was suspended due to the COVID-19 pandemic. For this study, we use waves 2016, 2017, 2018, 2019, 2022, and 2023. The 2021 wave is excluded because the questionnaire version omitted key variables for the analysis. By wave 6, cumulative panel attrition was approximately 40%; we therefore restrict the analysis to a balanced panel, yielding a two-level data set of *N* = 5,755 observations nested within *N* = 1,027 individuals. Additional documentation on sampling, attrition, and weighting is available from the ELSOC study team, and the data are publicly accessible through the ELSOC Dataverse.

### 3.2 Variables

#### 3.2.1 Market justice preferences for pensions

The dependent variable in this study is preferences for market justice in the pension system. This construct is measured by a single item that captures how strongly individuals endorse conditioning access to better pensions on income. Specifically, the question is: “Is it fair that high-income individuals have better pensions than lower-income individuals?” The response options range from 1 (“Strongly disagree”) to 5 (“Strongly agree”), so that higher values indicate stronger support for market-justified pension inequality.

#### 3.2.2 Social class

As for the independent variables, a class scheme was constructed based on that of Erikson, Goldthorpe, and Portocarero (EGP) (Erikson and Goldthorpe, 1992b), collapsed into three categories: (1) Working class (V+VI+VII), which serves as the reference category, (2) Intermediate class (III+IV), and (3) Service class (I+II). Additionally, a residual category is included that considers people outside the labor force according to their statements in the survey.

We employ the EGP scheme because it captures theoretically relevant dimensions of labor market position for understanding distributive justice attitudes. Unlike occupational prestige scales or purely income-based measures, EGP distinguishes positions based on employment relations and market situations. Such situations are the degree of autonomy, job security, and contractual conditions (Barone et al., 2022; Barozet et al., 2021) that structure both contributions to the pension system and expected returns. This distinction is crucial for our analysis as different class positions imply distinct relationships with pension schemes: service class workers typically benefit from stable employment and higher contribution rates, while working class positions face greater employment instability and lower coverage. The collapsed three-category version balances theoretical meaningfulness with statistical power, maintaining the core distinction between labor contract types (labor contracts for the working class, service relationships for the service class, and mixed positions for the intermediate class) while ensuring adequate sample sizes for longitudinal analysis.

The conversion from ISCO-08 occupational codes to EGP categories was performed using the *occupar* R package, which incorporates information on employment status (self-employed versus employee) and number of employees for self-employed workers. This approach follows the theoretical logic that class position depends not only on occupational title but also on employment relations, allowing us to differentiate, for example, between small-scale self-employed workers (petty bourgeoisie, class IVc) and employers with larger workforces (class I-II). A recognized limitation in contexts like Chile is EGP's difficulty in adequately capturing labor informality, as it was designed for formalized labor markets. However, this limitation is shared by most available occupational classification schemes and does not invalidate its analytical utility for the formal sector, which constitutes the base of the contributory pension system under normative evaluation in our dependent variable.

#### 3.2.3 Meritocracy

Following Young's (1975) distinction, this construct is operationalized into two dimensions: effort and talent. Both dimensions are measured by the agreement with statements indicating that effort and talent are rewarded in society. Specifically, meritocratic perceptions of effort are captured by the item “In Chile, people are rewarded for their efforts” (Merit: Effort), whereas meritocratic perceptions of talent by the item “In Chile, people are rewarded for their intelligence and abilities” (Merit: Talent). Both items are answered on a Likert scale ranging from 1 (“Strongly disagree”) to 5 (“Strongly agree”), with higher values indicating stronger perceptions that Chilean society operates according to meritocratic principles.

Table 1 shows the main variables of the study, their response categories and their frequencies.

**Table 1:** Variable descriptive statistics for the last wave (2023, *N* = 1.694).

**Label**	**Stats/values**	**Freqs (% of Valid)**	**Valid**
Pension distributive justice	1. Strongly disagree	296 (17.5%)	1,694
	2. Disagree	687 (40.6%)	(100.0%)
	3. Neither agree nor disagre	235 (13.9%)	
	4. Agree	419 (24.7%)	
	5. Strongly agree	57 (3.4%)	
Social class	1. Service class (I+II)	146 (8.6%)	1,694
	2. Intermediate class (III+I)	459 (27.1%)	(100.0%)
	3. Working class (V+VI+VII)	421 (24.9%)	
	4. Out of labor force	668 (39.4%)	
People are rewarded for their efforts	1. Strongly disagree	174 (10.3%)	1,694
	2. Disagree	776 (45.8%)	(100.0%)
	3. Neither agree nor disagre	379 (22.4%)	
	4. Agree	335 (19.8%)	
	5. Strongly agree	30 (1.8%)	
People are rewarded for their intelligence	1. Strongly disagree	142 (8.4%)	1,694
	2. Disagree	676 (39.9%)	(100.0%)
	3. Neither agree nor disagre	429 (25.3%)	
	4. Agree	418 (24.7%)	
	5. Strongly agree	29 (1.7%)	

#### 3.2.4 Controls

Based on previous research (Castillo et al., 2025; Jaime-Castillo, 2013; Lindh, 2015), the statistical models include sociodemographic and attitudinal controls to account for potential compositional effects in the population. We adjust for sex (1 = male, 2 = female), age (in years), educational attainment (1 = less than university, 2 = university), and political identification (1 = left, 2 = center, 3 = right, 4 = no identification). Following Langsåther et al. (2022), we do not control for income, as it can be conceived as a consequence of social class —that is, a post-treatment variable—and thus risks introducing collider bias into the estimates. Descriptive statistics for the control variables can be found in the Supplementary material.

### 3.3 Analytical strategy

To examine longitudinal changes in pension inequality justification and its relationship with social class and meritocratic beliefs, we employ Cumulative Link Mixed Models (CLMM) for ordinal responses. This approach is particularly well-suited for our analytical goals for several reasons. First, it preserves the ordinal nature of our dependent variable without imposing assumptions about equal Numerical distance between response categories. Second, it accounts for the hierarchical structure of our panel data, with repeated observations (Level 1) nested within individuals (Level 2). Third, it enables the decomposition of variance into within-person (WE) and between-person (BE) components, allowing us to distinguish temporal changes within individuals from stable differences between individuals (Bell et al., 2019; Singer, 2003).

#### 3.3.1 Within-person and between-person random effects models

Following the recommendations of Bell and Jones (2015), we decompose time-varying predictors into their within-person and between-person components using person-mean centering:


$$\begin{array}{l} X_{WE_{it}}=X_{it}-{\bar{X}}_{i}\\ \;X_{BE_{i}}={\bar{X}}_{i} \end{array}$$

where *X*_*it*_ represents the observed value for individual *i* at time *t*, ${\bar{X}}_{i}$ is the person-specific mean across all observations, *X*_*W*_*E*__*it*__ captures within-person deviations from the individual mean (representing temporal fluctuations), and *X*_*B*_*E*__*i*__ represents stable between-person differences (time-invariant characteristics).

This decomposition allows us to distinguish two theoretically distinct processes: (1) Within-person effects capture how changes in an individual's meritocratic beliefs over time relate to changes in their pension inequality justification, controlling for stable individual characteristics. (2) Between-person effects capture how individuals with different average levels of meritocratic beliefs differ in their pension inequality justification, net of temporal fluctuations.

#### 3.3.2 Model specification

The general form of our cumulative link mixed model is:


$$\begin{array}{l} \;\eta_{it}=\beta_{0}+\beta_{1}{\text{time}}_{it}+\beta_{2}Merit_{WE_{it}}+\beta_{3}X_{BE_{i}}+\beta_{4}{\text{Class}}_{i}\\ +\beta_{5}\left({\text{Class}}_{i}\times X_{WE_{it}}\right)+\beta_{6}\left({\text{Class}}_{i}\times X_{BE_{i}}\right)+u_{0i}+u_{1i}{\text{time}}_{it} \end{array}$$

where η_*it*_ is the linear prediction for individual *i* at time *t*, linked to the cumulative probabilities through a logit link function; β_0_ is the fixed intercept (threshold); β_1_ captures the linear time trend; β_2_ and β_3_ represent within-person and between-person effects of meritocratic perceptions, respectively; β_4_ captures social class between effects; β_5_ and β_6_ represent interactions between social class and within/between-person meritocratic perceptions; *u*_0*i*_ is the random intercept (capturing unobserved individual heterogeneity); and *u*_1*i*_ is a random slope for time (allowing individual-specific temporal trajectories).

The random effects are assumed to follow a bivariate normal distribution:


$$\begin{array}{l} \left(\begin{array}{c} u_{0i}\\ u_{1i} \end{array}\right)\sim N\left(\begin{array}{c} \left(\begin{array}{c} 0\\ 0 \end{array}\right),\left(\begin{array}{cc} \sigma_{u_{0}}^{2} & \sigma_{u_{01}}\\ \sigma_{u_{01}} & \sigma_{u_{1}}^{2} \end{array}\right) \end{array}\right) \end{array}$$

#### 3.3.3 Model building strategy

We estimate a series of nested models to test our hypotheses:

1. **Model 1 (time effects)**: Baseline model examining temporal trends with linear quadratic terms. First as categorical and then as continuous.
2. **Model 2 (class effects)**: Adds social class as a predictor
3. **Model 3 (within-person effects)**: Includes within-person meritocratic beliefs.
4. **Model 4 (between-person effects)**: Adds between-person meritocratic beliefs.
5. **Model 5 (full model with controls)**: Incorporates sociodemographic controls (education, political identification, sex, age).
6. **Model 6 (interactions)**: Tests interactions between social class and meritocratic beliefs (both WE and BE).

All models are estimated using maximum likelihood with the Hessian matrix computed explicitly to obtain standard errors. Models are implemented using the clmm() function from the ordinal package in R (Haubo and Christensen, 2018).

## 4 Results

### 4.1 Descriptive analysis

**Figure 1:**
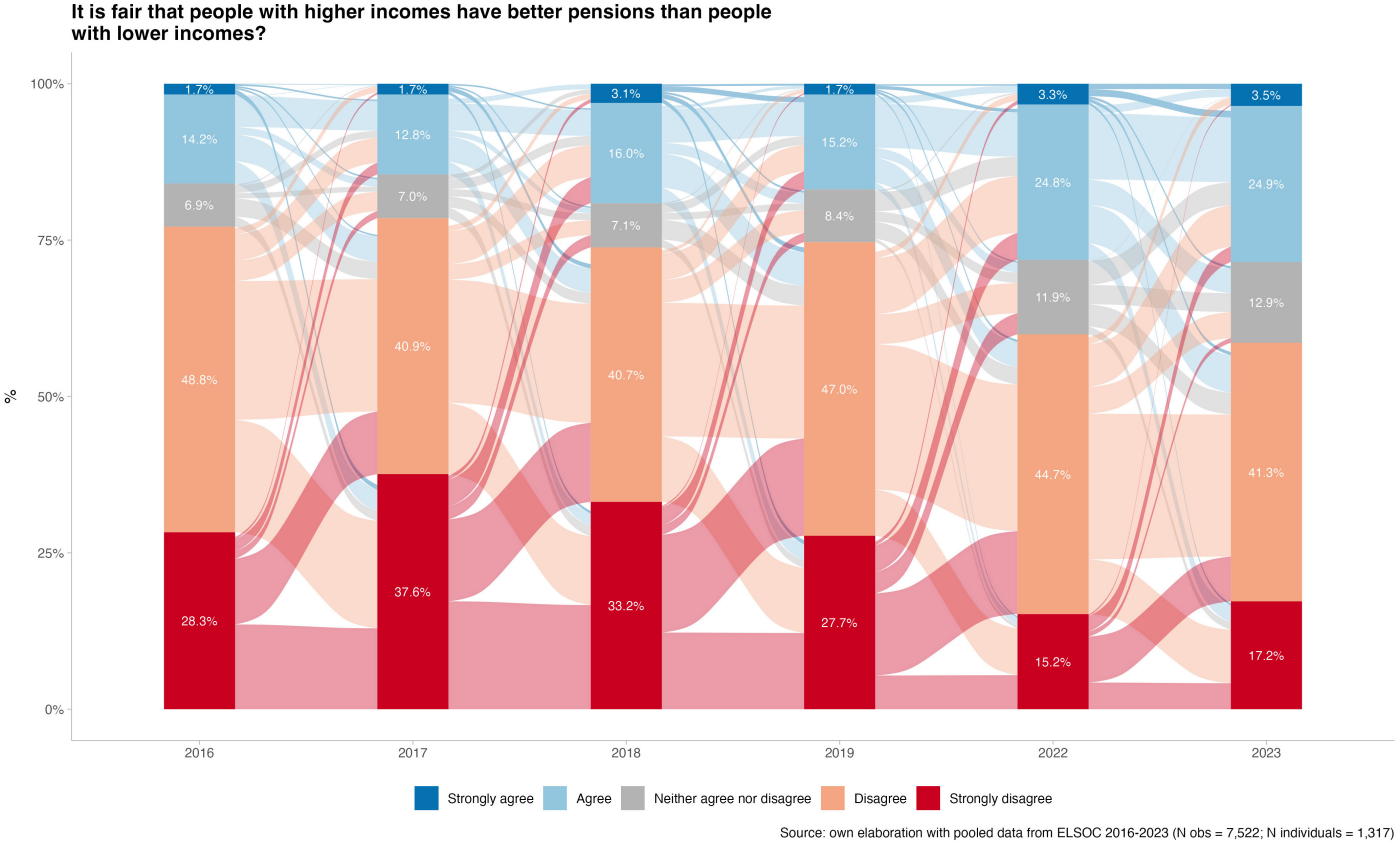
Changes in preferences for pension market justice over time (2016–2023). Source: own elaboration with pooled data from ELSOC 2016–2023 (N obs = 7,522; N individuals = 1,317).

Figure 1 depicts the temporal evolution of market justice preferences in pensions from 2016 to 2023. Each year presents percentage frequencies, and the flows between them reflect changes in opinion among the same people from one year to the next. The results indicate substantial attitudinal fluidity, with extensive cross-flows suggesting that many respondents revise their views over time rather than holding fixed positions. Most changes occur between adjacent categories, consistent with gradual shifts, although some movements span multiple categories. For example, among those who strongly disagreed with income-based pension access in 2019, about 15.2% remained in that position by 2022, while the remaining 12.5% shifted to other categories, primarily to disagreement or agreement. Overall, the majority continue to disagree with the notion that it is fair for higher-income people to receive better pensions than those with lower incomes. Yet, agreement has increased over time: the combined share of agree + strongly agree rises from 16.9% in 2019 to 28.4% in 2023 (an increase of roughly 11.5 percentage points). Transitions appear particularly turbulent during the 2019–2022 period—overlapping with social unrest and the pandemic—suggesting that these attitudes would be responsive to major contextual shocks and shifts in pension-related public discourse.

### 4.2 Bivariate relationships

**Figure 2:**
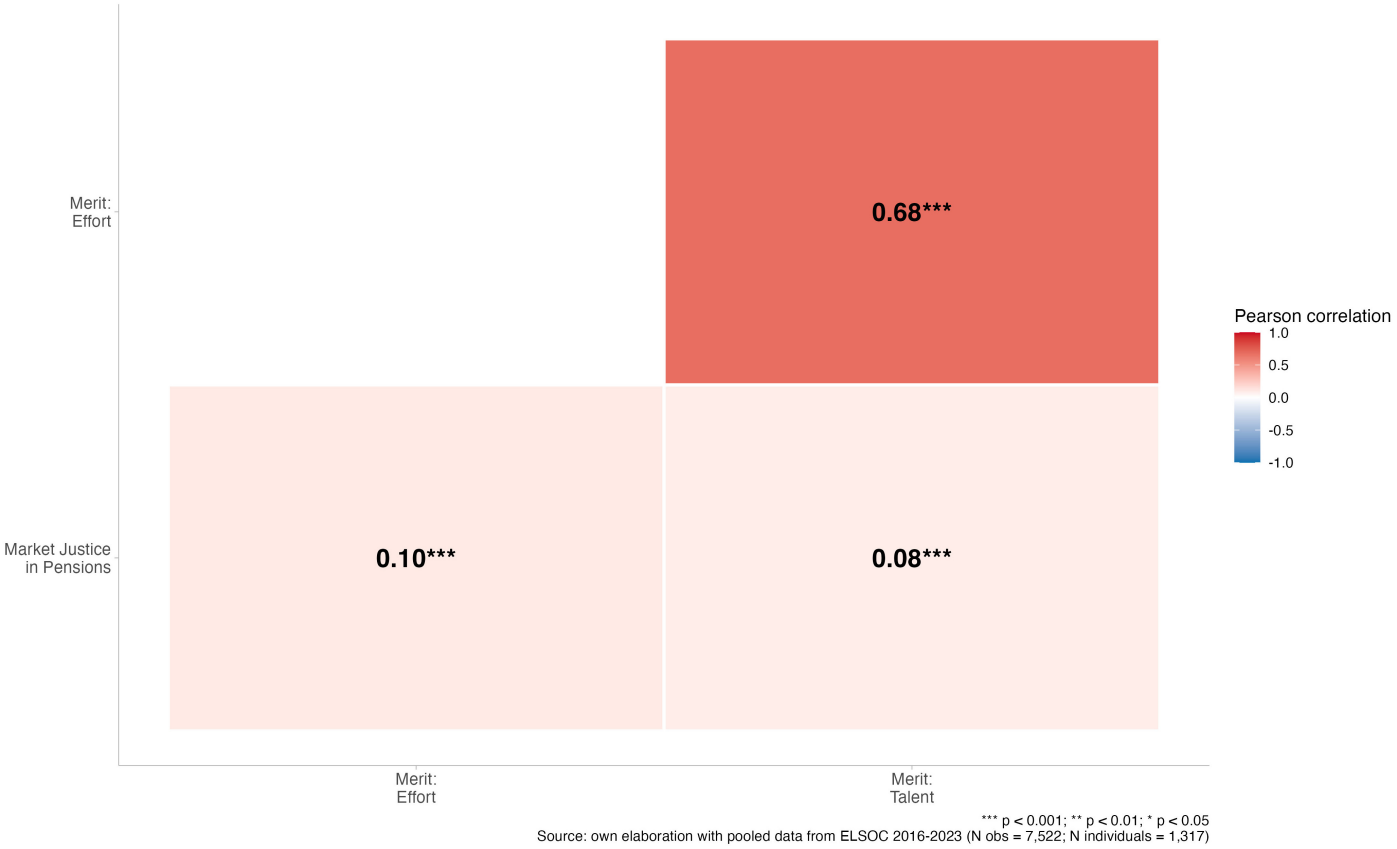
Correlation matrix between meritocratic perceptions and market justice preferences in pension. ^***^p < 0.001; ^**^p < 0.01; ^*^p < 0.05. Source: own elaboration with pooled data from ELSOC 2016–2023 (N obs = 7,522; N individuals = 1,317).

Figure 2 shows the correlation matrix between meritocratic perceptions and preferences for pension market justice. Both effort-based (*r* = 0.10, *p* < 0.001) and talent-based (*r* = 0.08, *p* < 0.001) meritocratic perceptions are positively associated with greater support for market-based pension welfare. Although these correlations are modest, their statistical significance indicates a consistent bivariate relationships. The two meritocracy indicators are also strongly correlated (*r* = 0.68, *p* < 0.001), suggesting they capture a shared meritocratic worldview while remaining sufficiently distinct to justify treating them as separate constructs. Taken together, these patterns provide initial support for the expectation that meritocratic perceptions are associated with greater acceptance of income-linked pension outcomes, while also suggesting that other factors likely account for most of the variation in these preferences.

**Figure 3:**
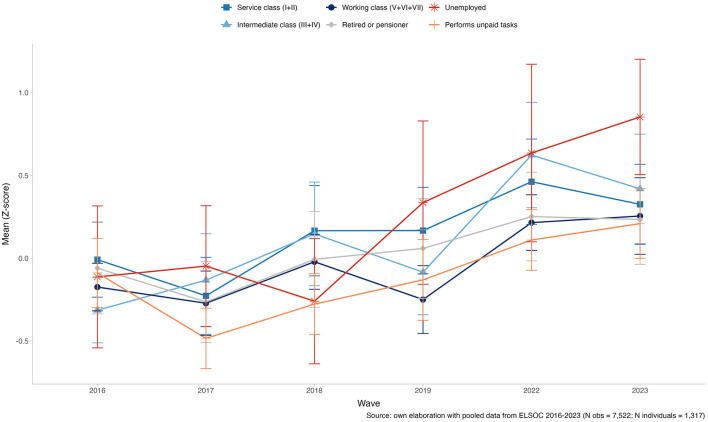
Changes in the standarized mean of market justice preferences in pensions by social class (2016–2023). Source: own elaboration with pooled data from ELSOC 2016–2023 (N obs = 7,522; N individuals = 1,317).

Turning to social class differences, Figure 3 plots longitudinal trajectories of pension market justice by class from 2016 to 2023. Overall, the descriptive trends suggest that there are no differences between social classes in their level of preference for market justice in pensions. The figure shows a broadly upward trend across all classes, especially after 2019, alongside increasingly similar levels over time. The unemployed group shows the most significant change, moving from strong rejection (≈0.6 in 2016) to moderate acceptance (≈0.7 in 2023). Retirees/pensioners also increase steadily, from near zero in 2016 to roughly 0.5 by 2023, and the service class rises notably—particularly between 2019 and 2022—reaching values close to 0.5. The working class follows a similar trajectory, though at slightly lower levels. Overall, the trajectories suggest convergence toward greater acceptance of pension inequality during 2019–2023, a period marked by social unrest, the pandemic, and pension fund withdrawals (*10% retiros*), raising questions about how these disruptions and the surrounding policy debate reshaped views of distributive justice.

### 4.3 Multilevel models

Table 2 reports estimates from the cumulative longitudinal multilevel models of support for pension market justice, distinguishing effects operating between individuals (BE) and within individuals over time (WE). The intraclass correlation coefficient (Hox et al., 2017) from the null model (see Supplementary material), which partitions the total variance in the outcome, is 0.24. This implies that about 24% of the variance lies between individuals, while the remaining 76% reflects within-person change across survey waves.

**Table 2:** Cumulative link longitudinal multilevel models for pension market justice preferences.

	**Model 1**	**Model 2**	**Model 3**	**Model 4**	**Model 5**	**Model 6**
**Wave (Ref. = 2016)**						
Wave 2017	−0.337^***^ (0.079)					
Wave 2018	−0.013 (0.078)					
Wave 2019	0.086 (0.077)					
Wave 2022	0.879^***^ (0.078)					
Wave 2023	0.854^***^ (0.077)					
Wave		−0.182^**^ (0.064)	−0.179^**^ (0.064)	−0.187^**^ (0.064)	−0.190^**^ (0.064)	−0.192^**^ (0.064)
Wave^2^		0.059^***^ (0.009)	0.058^***^ (0.009)	0.060^***^ (0.009)	0.060^***^ (0.009)	0.060^***^ (0.009)
**Social class (Ref. = Working class (V+VI+VII)**						
Intermediate class (III+IV)			0.096 (0.120)	0.101 (0.121)	0.147 (0.118)	0.185 (0.117)
Service class (I+II)			0.169 (0.108)	0.169 (0.108)	0.158 (0.106)	0.017 (0.109)
Retired or pensioner			0.103 (0.117)	0.096 (0.117)	0.030 (0.115)	0.110 (0.127)
Unemployed			0.101 (0.158)	0.105 (0.158)	0.147 (0.155)	0.119 (0.152)
Performs unpaid tasks			−0.199 (0.110)	−0.198 (0.111)	−0.208 (0.109)	0.029 (0.113)
Merit: Effort (WE)				0.114^**^ (0.035)	0.117^***^ (0.035)	0.115^**^ (0.035)
Merit: Talent (WE)				0.066 (0.034)	0.066 (0.034)	0.067 (0.034)
Merit: Effort (BE)					0.391^***^ (0.097)	0.387^***^ (0.093)
Merit: Talent (BE)					0.079 (0.097)	0.028 (0.094)
Controls	No	No	No	No	No	Yes
BIC	19, 107.264	19, 166.031	19, 199.863	19, 182.632	19, 144.360	19, 097.183
Numb. obs.	7, 522	7, 522	7522	7, 522	7, 522	7, 522
Num. groups: individuals	1, 317	1, 317	1, 317	1, 317	1, 317	1, 317
Var: individuals (Intercept)	1.151	1.334	1.302	1.288	1.146	1.014
Var: individuals, wave		0.018	0.018	0.015	0.015	0.016

Cells contain regression coefficients with standard errors in parentheses. ^***^p < 0.001; ^**^p < 0.01; ^*^p < 0.05.

Model 1, which adds survey-wave indicators to capture temporal shifts in the outcome, shows a decline in 2017 (β = –0.337, *p* < 0.001) relative to 2016. By contrast, the 2022 and 2023 waves exhibit significant increases in market justice preferences for pension (β = 0.879, *p* < 0.001; β = 0.854, *p* < 0.001), indicating a nonlinear pattern over time. To account for this trajectory, Model 2 specifies time (survey wave) as a continuous predictor and includes a quadratic term to capture the curvature suggested by Model 1. The negative linear coefficient indicates an overall downward trend in market-based access to old-age pension benefits, whereas the positive quadratic term signals a subsequent upturn in the final measurement points.

Model 3 adds between-group effects (BE) for social class, capturing differences in average support for pension market justice across class locations. Overall, the estimates provide no evidence of systematic class differences in the support for market-based access to old-age pension benefits. Using the working class as the reference group, those in the intermediate class (β = 0.10, *p* > 0.05), the service class (β = 0.17, *p* > 0.05), retirees (β = 0.10, *p* > 0.05), and the unemployed (β = 0.10, *p* > 0.05) exhibit positive coefficients, but none reach conventional levels of statistical significance. Likewise, individuals performing unpaid work (e.g., domestic or care work) show a negative coefficient relative to the working class, which is not statistically significant (β = –0.20, *p* > 0.05). Taken together, these results suggest that neither class position nor being outside the labor force is clearly associated with differences in preferences for pension market justice at the between-individual level.

Models 4 and 5 extend the analysis by incorporating the within-person (WE) and between-person (BE) components of the meritocratic variables. Model 4 examines how changes in individuals' meritocratic perceptions over time relate to their views on pension market justice. The estimates show that the within-effect of perceiving that effort is rewarded is positive and highly significant (*p* < 0.001). Holding all other predictors constant, a one-point increase in effort-based meritocratic perceptions for the same respondent between waves is associated with a 0.11-point increase in the support for market-based access to old-age pension benefits. Model 5, which captures between-person differences, points in the same direction. On average, respondents who see their society as more effort-meritocratic display stronger market-oriented views on pensions, with a 0.39-point higher score on the outcome, a statistically significant association. By contrast, the corresponding indicators for the perception that intelligence and ability are rewarded show no statistically significant association with the support for pension market justice at either the within (β = 0.07, *p* > 0.05)- or between-person level (β = 0.08, *p* > 0.05).

Model 6 introduces the control variables. The within- and between-effects of the main predictors remain stable in both sign and significance, indicating that the associations are robust (see Supplementary material for the effects of the control variables).

To examine whether meritocratic perceptions condition class differences in pension market justice preferences, we estimate four interaction models that combine between-person social class with the within- and between-person components of meritocratic perceptions. Overall, interaction terms are small and generally not statistically distinguishable from zero, and improvements in model fit over the baseline specifications are modest. This pattern indicates that the association between meritocratic perceptions and support for market-based pension allocation is similar across class locations (complete model estimates are reported in the Supplementary material).

**Figure 4:**
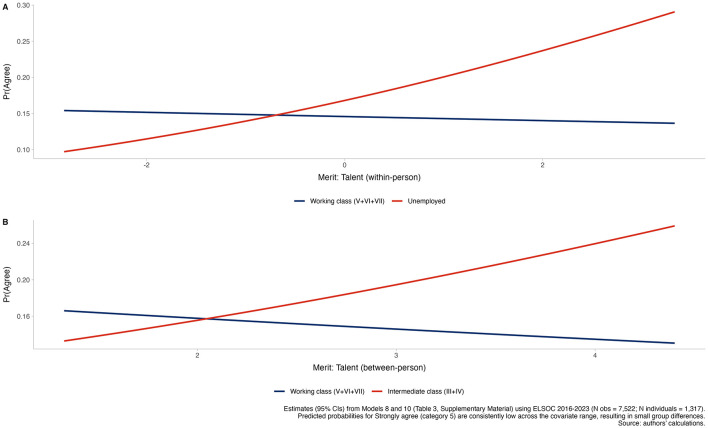
Predicted probabilities of pension market justice preferences by talent-based meritocratic perceptions and social class. Estimates (95% Cls) from Models 8 and 10 (Table 3, Supplementary material) using ELSOC 2016–2023 (N obs = 7,522; N individuals = 1,317). Predicted probabilities for strongly agree (category 5) are consistently low across the covariate range, resulting in small group differences. Source: authors' calculations.

Two interaction effects nevertheless stand out, which are illustrated in Figure 4. First, Figure 4A shows that the within-person association between talent-based meritocratic perceptions and pension market justice differs by labor-market status. Within-person deviations in perceived talent rewards are unrelated to pension market justice in the reference group (β = 0.024, *p* = 0.67), but become significantly more positive among unemployed respondents (interaction β = 0.257, *p* = 0.049), implying a net within-person effect of β = 0.233 (OR = 1.26). Second, Figure 4B indicates that the between-person association between average (person-mean) talent-based meritocratic perceptions and pension market justice varies by social class: it is null in the working class (β = 0.089, *p* = 0.50) but significantly stronger in the intermediate class (interaction β = 0.401, *p* = 0.035), yielding a positive between-person slope of β = 0.313 (OR = 1.37). Taken together, these models indicate a largely homogeneous association between meritocratic beliefs and pension market justice, with limited evidence of moderation. Where moderation emerges, it is concentrated in the talent dimension and confined to specific middle-class and non-class positions rather than reflecting systematic stratification across the whole class structure.

## 5 Discussion

Regarding descriptive trends, survey data from 2016 to 2023 reveal a persistent tension in Chileans' judgments regarding pension market justice. In every wave, clear majorities reject the idea that it is fair for higher-income individuals to receive better pensions than lower-income individuals. At the same time, a small—but expanding—segment endorses this income-based principle, with the sharpest growth occurring between 2018 and 2023. Pension market justice, therefore, remains a minority orientation, yet one that has become more prevalent over time, in line with longitudinal evidence of rising support for income-based access to other welfare services in Chile, such as healthcare and education (Castillo et al., 2025). These attitudes are formed within an institutional architecture of mandatory, privately managed, defined-contribution accounts that tightly link old-age income to labor earnings, contribution stability, and market performance (Madero-Cabib et al., 2019; Solimano, 2021), and that can symbolically recast pensions from solidarity-based social rights into individual financial assets. In this respect, Chile mirrors broader findings that income-based access to welfare is more strongly endorsed in liberal, marketised regimes (Lindh, 2015). Taken together, these descriptive patterns suggest that support for pension market justice—contested and far from hegemonic—nonetheless constitutes a salient orientation that invites explanation: which social trajectories and belief structures sustain it?

Concerning social class, the results provide no support for *H*_1_, which predicted stronger endorsement of pension market justice among the upper classes than among the lower classes. The longitudinal multilevel models indicate that neither the intermediate class nor the service class differs significantly from the working class in their pension market justice preferences. This null finding is substantively meaningful because it indicates that class position—despite being a fundamental structural determinant of pension outcomes in Chile's individual capitalization system (Gálvez et al., 2024; Madero-Cabib et al., 2019)—does not translate into systematic differences in attitudes toward the system's distributive logic. Moreover, the lack of significant class effects persists across most specifications, suggesting that this is a robust empirical pattern rather than an artifact of a particular model choice or set of controls.

This absence of class differentiation contradicts expectations derived from material interests (Lindh, 2015; Svallfors, 2006) and contrasts with evidence from developed welfare states (Busemeyer and Iversen, 2020; Lindh, 2015) and from market-oriented welfare configurations in Latin America (Kerner, 2020), where privileged groups tend to endorse market allocation more strongly than lower classes. The result is particularly striking given the system's sharply unequal material consequences across class positions: while privileged groups with stable formal employment can secure higher pensions through sustained contributions, working-class trajectories are more often precarious and yield insufficient retirement income (Cabib, 2025; Gil-Hernández et al., 2025). A plausible interpretation of this disconnection between objective class interests and subjective justice evaluations lies in the individualizing logic of Chile's capitalization system, which reframes distributive conflict away from class-based struggles and toward individualized responsibility (Arrizabalo Montoro et al., 2019). By presenting pensions as the direct outcome of personal contributions and choices, the system may obscure the extent to which “contributory capacity” itself reflects structural inequalities rather than individual merit, thereby weakening the attitudinal imprint of class position—even among those most disadvantaged by the market model.

This phenomenon of “class neutralization” contrasts sharply with the established findings of comparative research on welfare, in which class position tends to shape distributive preferences in predictable ways (Svallfors, 2006; Svallfors, 2007). While previous studies in both developed welfare states (Busemeyer and Iversen, 2020; Lindh, 2015) and in Latin American contexts (Kerner, 2020) document more market-oriented attitudes among privileged groups, our results suggest that decades of pension privatization may have eroded this class-based differentiation by incorporating individualized responsibility as the dominant normative framework.

Turning to meritocratic perceptions, *H*_2*a*_ and *H*_2*b*_ predicted that stronger meritocratic perceptions would be associated with greater support for market-based access to pensions. The results offer partial but revealing support for this hypothesis. At the between-person level (BE), the perception that effort is rewarded in society is strongly positively associated with pension market justice. In contrast, the perception that talent is rewarded is not statistically significant. At the within-person level (WE), effort-based meritocratic perceptions also show a significant positive association, albeit of smaller magnitude, whereas talent remains non-significant. Substantively, this means that individuals who, on average, perceive more substantial effort–reward linkages are more supportive of pension market justice, and individuals who increase their effort-based meritocratic perceptions over time also tend to increase their support for pension market justice. The contrast between effort and talent suggests that when people justify pension market allocation, they draw primarily on narratives of hard work and responsibility rather than on innate ability.

These findings align with a broader view of meritocracy as a legitimizing ideology in highly unequal contexts. In Chile's individual capitalization system, where pensions are institutionally presented as the direct result of personal contributions and choices, the effort narrative provides a compelling cognitive frame for justifying disparities: higher pensions can be interpreted as the deserved outcome of “working harder,” regardless of the structural constraints faced by different groups (Castillo et al., 2025). This pattern echoes scholarship suggesting that meritocratic perceptions can intensify precisely where real opportunities are most constrained (Harrs and Sterba, 2025; Mijs, 2021). Importantly, the explanatory power of effort-based perceptions also helps contextualize the null class pattern observed for *H*_1_: meritocratic ideology can offer a culturally available justification that renders unequal pension outcomes intelligible and acceptable, thereby dampening the extent to which class position translates into divergent justice evaluations.

Against this backdrop, a primary interest of the study was to examine whether meritocratic perceptions condition class differences in pension market justice (*H*_3*a*_ and *H*_3*b*_), with more substantial meritocratic effects expected among higher classes. The results point to a more selective and class-specific pattern than initially hypothesized. Rather than a generalized moderation among the service class, meritocratic perceptions—particularly talent-based ones—operate primarily among the intermediate class. At the between-person level (*H*_3*a*_), this class exhibits a significant positive interaction with talent-based meritocratic perceptions, indicating that intermediate-class individuals who hold stronger and more stable beliefs that talent is rewarded are more likely to support pension commodification. In contrast, no comparable interaction emerges for effort, and no equivalent moderation appears within the service class. One interpretation is that those in the most advantaged positions may have less need to rely on talent-based justifications because their outcomes can be plausibly narrated through dominant accounts of sustained effort, credentials, and accumulated advantages (Lindh and McCall, 2020). The within-person analysis (*H*_3*b*_) further qualifies the moderation story: intra-individual changes in meritocratic perceptions do not generally moderate the relationship between class and pension market justice for either effort or talent, suggesting that short-term fluctuations are insufficient to reshape class-linked orientations and that what matters more are stable, long-term orientations—particularly toward talent as a distributive principle—within specific class locations.

An important exception, however, emerges for unemployed respondents. Within-person deviations in perceived talent rewards are unrelated to pension market justice in the working class, but become significantly more positive among the unemployed. Substantively, this indicates that among unemployed individuals, increases over time in the perception that talent is rewarded are associated with higher support for pension market justice. A possible interpretation of this caveat is that unemployment weakens the experiential plausibility of effort-based narratives. When one's effort is not being translated into employment or stable contributions, talent may become a more salient explanatory lens for unequal outcomes in core welfare domains such as pensions. In this sense, the unemployed may be especially prone to shifting toward talent-based meritocratic accounts to explain distributive differences, and these shifts, in turn, are associated with greater endorsement of market-based pension justice.

Taken together, these findings suggest that in a context where effort does not reliably translate into secure welfare outcomes—such as Chile's privatized, individual-contribution pension system—the intermediate class may more readily draw on talent-based explanations to legitimize modest relative advantages and to rationalize why increased effort does not necessarily yield service-class outcomes (Castillo et al., 2025; Lindh and McCall, 2020). The service class, by contrast, appears less dependent on this symbolic reinforcement, as its privileged position can be more coherently explained through widely shared narratives of effort and qualifications.

## 6 Conclusion

This article examined the complex interaction between social class, meritocratic perceptions, and preferences for market justice in the Chilean pension system during the period between 2016 and 2023. Using longitudinal data from the ELSOC survey, we explored how the individual capitalization model—a system that links old-age benefits to personal contributions—shapes the normative foundations of social justice. By analyzing these dynamics during a period marked by significant social unrest and political discussions around a new constitution, the study offers a critical view of how market logic and subjective beliefs about equity coexist in a highly unequal and commodified environment.

The findings reveal a paradox where objective social class does not significantly predict attitudes toward pension market justice; instead, support for income-based pension differences grew across all strata, reaching nearly 28.4% by 2023. This suggests that the individualizing logic of the pension system has succeeded in reframing distributive conflicts as matters of personal responsibility, leading to a generalized pattern of beliefs across classes and supporting the idea of markets as moral projects (Fourcade and Healy, 2007). Meritocratic perceptions, specifically those emphasizing individual effort, were found to be the primary drivers of this support, acting as a powerful moral justification that allows individuals to legitimize pension disparities regardless of their own structural disadvantages. While effort-based beliefs remained stable predictors of fairness, talent-based justifications emerged more selectively, particularly within the intermediate class, as a tool to rationalize modest relative advantages.

This research advances on previous work by shifting the focus from objective socio-economic measures to the longitudinal, subjective interplay of class and meritocratic ideology. Unlike previous studies that assume material interests automatically dictate policy preferences, this work emphasizes how a “policy feedback” mechanism in a privatized regime can erode class-based demands for redistribution. The study underscores how the Chilean model symbolically recasts social rights as financial assets, illustrating the resilience of meritocratic narratives even in the face of widespread public discontent and systemic inequality.

In terms of pathways for future research, international comparative studies are needed to determine whether this ‘class neutralization' is unique to Chile's neoliberal history or whether it is a universal feature of privatized welfare regimes. Future studies should also explore the role of political communication, and discussions on pensions funds withdrawals during the pandemic, on changing perceptions of pension ownership and fairness. Also, investigating how these normative beliefs translate into behavioral—as voting patterns or participation in social movements such as “No+AFP”—would provide valuable insights into the path from subjective perceptions to actual policy change. Finally, it is also important to consider how to integrate new measures of meritocracy (Castillo et al., 2023) into future studies, as this could reveal other configurations of beliefs between preferences for market justice and merit that the current operationalization does not allow to observe.

## Data Availability

Publicly available datasets were analyzed in this study. This data can be found here: https://dataverse.harvard.edu/api/access/datafile/10797987.
