# Supplementary material for: Justification trajectories for pension inequality in Chile (2016–2023): the role of social class and beliefs in meritocracy
Source: Front Sociol. 2026 Mar 10;11:1771856. doi: 10.3389/fsoc.2026.1771856 (PMC13008653; doi:10.3389/fsoc.2026.1771856)
Supplement: Supplementary file 1 [file Data_Sheet_1.pdf]

Supplementary material

1 Descriptive statistics for control variables

Table 1: Descriptive statistics for control variables for the last wave (2023)

Label	Stats / Values	Freqs (% of Valid)	Valid
Educational level	1. Less than Universitary<br>2. Universitary	1090 (83.9%)<br>209 (16.1%)	1299<br>(100.0%)
Sex	1. Male<br>2. Female	433 (33.3%)<br>866 (66.7%)	1299<br>(100.0%)
Age	Mean (sd) : 47.7 (14.3)<br>min < med < max:<br>18 < 48 < 78<br>IQR (CV) : 22 (0.3)	60 distinct values	1299<br>(100.0%)
Political identification	1. Left<br>2. Center<br>3. Right<br>4. Does not identify	272 (20.9%)<br>263 (20.2%)<br>176 (13.5%)<br>588 (45.3%)	1299<br>(100.0%)

2 Complete longitudinal multilevel models

Table 2: Complete cumulative longitudinal multilevel models for pension market justice preferences

	Model 0	Model 1	Model 2	Model 3	Model 4	Model 5	Model 6
Strongly disagree|Disagree	-1.258^{***}	-1.062^{***}	-1.057^{***}	-1.022^{***}	-1.031^{***}	0.197	-0.063
	(0.041)	(0.066)	(0.106)	(0.119)	(0.119)	(0.208)	(0.250)
Disagree|Neither agree nor disagree	1.014^{***}	1.327^{***}	1.342^{***}	1.376^{***}	1.373^{***}	2.600^{***}	2.340^{***}
	(0.040)	(0.067)	(0.108)	(0.121)	(0.121)	(0.212)	(0.252)
Neither agree nor disagree|Agree	1.606^{***}	1.948^{***}	1.968^{***}	2.002^{***}	1.999^{***}	3.226^{***}	2.967^{***}
	(0.043)	(0.070)	(0.110)	(0.123)	(0.123)	(0.214)	(0.254)
Agree|Strongly agree	4.166^{***}	4.582^{***}	4.616^{***}	4.652^{***}	4.650^{***}	5.878^{***}	5.620^{***}
	(0.085)	(0.103)	(0.136)	(0.146)	(0.146)	(0.231)	(0.268)
Wave (Ref.= 2016)							
Wave 2017		-0.337^{***}					
		(0.079)					
Wave 2018		-0.013					
		(0.078)					
Wave 2019		0.086					
		(0.077)					
Wave 2022		0.879^{***}					
		(0.078)					
Wave 2023		0.854^{***}					
		(0.077)					
Wave			-0.182^{**}	-0.179^{**}	-0.187^{**}	-0.190^{**}	-0.192^{**}
			(0.064)	(0.064)	(0.064)	(0.064)	(0.064)
Wave^2			0.059^{***}	0.058^{***}	0.060^{***}	0.060^{***}	0.060^{***}
			(0.009)	(0.009)	(0.009)	(0.009)	(0.009)
Social class (Ref.= Working class (V+VI+VII)							
Intermediate class (III+IV)				0.096	0.101	0.147	0.185
				(0.120)	(0.121)	(0.118)	(0.117)
Service class (I+II)				0.169	0.169	0.158	0.017
				(0.108)	(0.108)	(0.106)	(0.109)
Retired or pensioner				0.103	0.096	0.030	0.110
				(0.117)	(0.117)	(0.115)	(0.127)
Unemployed				0.101	0.105	0.147	0.119
				(0.158)	(0.158)	(0.155)	(0.152)
Performs unpaid tasks				-0.199	-0.198	-0.208	0.029
				(0.110)	(0.111)	(0.109)	(0.113)
Merit: Effort (WE)					0.114^{**}	0.117^{***}	0.115^{**}
					(0.035)	(0.035)	(0.035)
Merit: Talent (WE)					0.066	0.066	0.067
					(0.034)	(0.034)	(0.034)
Merit: Effort (BE)						0.391^{***}	0.387^{***}
						(0.097)	(0.093)
Merit: Talent (BE)						0.079	0.028
						(0.097)	(0.094)
Universitary education (Ref.= Less than Universitary)							0.540^{***}
							(0.105)
Political identification (Ref.= Left)							
Center							0.258^{*}
							(0.109)
Right							0.605^{***}
							(0.122)
Does not identify							0.152
							(0.093)
Female (Ref.= Male)							-0.493^{***}
							(0.081)
Age (in years)							-0.002
							(0.003)
BIC	19488.077	19107.264	19166.031	19199.863	19182.632	19144.360	19097.183
Numb. obs.	7522	7522	7522	7522	7522	7522	7522
Num. groups: individuals	1317	1317	1317	1317	1317	1317	1317
Var: individuals (Intercept)	1.024	1.151	1.334	1.302	1.288	1.146	1.014
Var: individuals, wave			0.018	0.018	0.015	0.015	0.016

Note: Cells contain regression coefficients with standard errors in parentheses. $^{***}p < 0.001$; $^{**}p < 0.01$; $^{*}p < 0.05$.

Table 3: Interactions for social class, meritocracy, and pension market justice preferences

	Model 7	Model 8	Model 9	Model 10
Social class (Ref.= Working class (V+VI+VII) x Meritocracy (WE)				
Intermediate class (III+IV) x Merit: Effort (WE)	0.109			
	(0.100)			
Service class (I+II) x Merit: Effort (WE)	0.080			
	(0.089)			
Retired or pensioner x Merit: Effort (WE)	0.020			
	(0.091)			
Unemployed x Merit: Effort (WE)	0.087			
	(0.134)			
Performs unpaid tasks x Merit: Effort (WE)	0.033			
	(0.092)			
Intermediate class (III+IV) x Merit: Talent (WE)		0.171		
		(0.099)		
Service class (I+II) x Merit: Talent (WE)		0.091		
		(0.089)		
Retired or pensioner x Merit: Talent (WE)		0.050		
		(0.091)		
Unemployed x Merit: Talent (WE)		0.257^{*}		
		(0.131)		
Performs unpaid tasks x Merit: Talent (WE)		0.174		
		(0.092)		
Social class (Ref.= Working class (V+VI+VII) x Meritocracy (BE)				
Intermediate class (III+IV) x Merit: Effort (BE)			0.390	
			(0.207)	
Service class (I+II) x Merit: Effort (BE)			0.141	
			(0.180)	
Retired or pensioner x Merit: Effort (BE)			0.217	
			(0.191)	
Unemployed x Merit: Effort (BE)			0.175	
			(0.256)	
Performs unpaid tasks x Merit: Effort (BE)			−0.062	
			(0.176)	
Intermediate class (III+IV) x Merit: Talent (BE)				0.386^{*}
				(0.196)
Service class (I+II) x Merit: Talent (BE)				0.210
				(0.178)
Retired or pensioner x Merit: Talent (BE)				0.119
				(0.187)
Unemployed x Merit: Talent (BE)				0.147
				(0.262)
Performs unpaid tasks x Merit: Talent (BE)				−0.141
				(0.179)
Controls	Yes	Yes	Yes	Yes
BIC	19167.451	19152.429	19170.659	19170.820
Numb. obs.	7522	7522	7522	7522
Num. groups: individuals	1317	1317	1317	1317
Var: individuals (Intercept)	1.112	1.116	2.507	1.634
Var: individuals, wave	0.014	0.013	0.016	0.019
Var: individuals, merit effort cwc	0.112			
Var: individuals, merit talent cwc		0.142		
Var: individuals, merit effort mean			0.329	
Var: individuals, merit talent mean				0.184

Note: Cells contain regression coefficients with standard errors in parentheses. $^{***}p < 0.001$; $^{**}p < 0.01$; $^{*}p < 0.05$. CWC = centered within group.

3 Robustness check

Table 4: Cumulative longitudinal multilevel models for pension market justice preferences with unbalanced data

	Model 0	Model 1	Model 2	Model 3	Model 4	Model 5	Model 6
Strongly disagree|Disagree	-1.177^{***}	-0.964^{***}	-0.981^{***}	-0.922^{***}	-0.925^{***}	0.190	0.091
	(0.026)	(0.046)	(0.075)	(0.082)	(0.083)	(0.127)	(0.152)
Disagree|Neither agree nor disagree	1.056^{***}	1.375^{***}	1.358^{***}	1.416^{***}	1.423^{***}	2.537^{***}	2.435^{***}
	(0.026)	(0.047)	(0.077)	(0.084)	(0.084)	(0.130)	(0.155)
Neither agree nor disagree|Agree	1.678^{***}	2.024^{***}	2.008^{***}	2.066^{***}	2.075^{***}	3.188^{***}	3.087^{***}
	(0.029)	(0.049)	(0.079)	(0.085)	(0.086)	(0.132)	(0.156)
Agree|Strongly agree	4.173^{***}	4.582^{***}	4.568^{***}	4.626^{***}	4.640^{***}	5.754^{***}	5.657^{***}
	(0.059)	(0.072)	(0.096)	(0.102)	(0.102)	(0.144)	(0.167)
Wave (Ref.= 2016)							
Wave 2017		-0.245^{***}					
		(0.057)					
Wave 2018		-0.007					
		(0.054)					
Wave 2019		0.123^{*}					
		(0.054)					
Wave 2022		0.941^{***}					
		(0.058)					
Wave 2023		0.880^{***}					
		(0.057)					
Wave			-0.178^{***}	-0.176^{***}	-0.182^{***}	-0.189^{***}	-0.191^{***}
			(0.045)	(0.045)	(0.045)	(0.045)	(0.045)
Wave^2			0.058^{***}	0.057^{***}	0.059^{***}	0.060^{***}	0.060^{***}
			(0.006)	(0.006)	(0.006)	(0.006)	(0.006)
Social class (Ref.= Working class (V+VI+VII)							
Intermediate class (III+IV)				0.084	0.084	0.118	0.113
				(0.073)	(0.073)	(0.072)	(0.071)
Service class (I+II)				0.276^{***}	0.277^{***}	0.268^{***}	0.126
				(0.062)	(0.062)	(0.061)	(0.065)
Retired or pensioner				0.090	0.091	0.032	0.124
				(0.071)	(0.071)	(0.070)	(0.079)
Unemployed				-0.019	-0.021	0.014	0.001
				(0.098)	(0.099)	(0.097)	(0.096)
Performs unpaid tasks				-0.163^{*}	-0.161^{*}	-0.167^{*}	0.002
				(0.070)	(0.070)	(0.069)	(0.072)
Merit: Effort (WE)					0.131^{***}	0.133^{***}	0.133^{***}
					(0.025)	(0.025)	(0.025)
Merit: Talent (WE)					0.093^{***}	0.094^{***}	0.094^{***}
					(0.025)	(0.025)	(0.025)
Merit: Effort (BE)						0.306^{***}	0.289^{***}
						(0.053)	(0.051)
Merit: Talent (BE)						0.120^{*}	0.088
						(0.052)	(0.050)
Universitary education (Ref.= Less than Universitary)							0.454^{***}
							(0.062)
Political identification (Ref.= Left)							
Center							0.291^{***}
							(0.063)
Right							0.634^{***}
							(0.071)
Does not identify							0.206^{***}
							(0.058)
Female (Ref.= Male)							-0.331^{***}
							(0.049)
Age (in years)							-0.002
							(0.002)
BIC	40997.788	40268.045	40385.873	40394.900	40305.889	40190.278	40052.228
Numb. obs.	15724	15724	15724	15724	15724	15724	15724
Num. groups: individuals	3784	3784	3784	3784	3784	3784	3784
Var: individuals (Intercept)	0.924	1.033	1.192	1.164	1.156	1.039	0.919
Var: individuals, wave			0.012	0.012	0.008	0.008	0.009

Note: Cells contain regression coefficients with standard errors in parentheses. $^{***}p < 0.001$; $^{**}p < 0.01$; $^{*}p < 0.05$.

Table 5: Interactions for social class, meritocracy, and pension market justice preferences with unbalanced data

	Model 7	Model 8	Model 9	Model 10
Social class (Ref.= Working class (V+VI+VII) x Meritocracy (WE)				
Intermediate class (III+IV) x Merit: Effort (WE)	0.085			
	(0.069)			
Service class (I+II) x Merit: Effort (WE)	−0.002			
	(0.061)			
Retired or pensioner x Merit: Effort (WE)	0.062			
	(0.066)			
Unemployed x Merit: Effort (WE)	0.165			
	(0.099)			
Performs unpaid tasks x Merit: Effort (WE)	0.070			
	(0.067)			
Intermediate class (III+IV) x Merit: Talent (WE)		0.139^{*}		
		(0.069)		
Service class (I+II) x Merit: Talent (WE)		0.043		
		(0.060)		
Retired or pensioner x Merit: Talent (WE)		0.051		
		(0.065)		
Unemployed x Merit: Talent (WE)		0.208^{*}		
		(0.097)		
Performs unpaid tasks x Merit: Talent (WE)		0.111		
		(0.066)		
Social class (Ref.= Working class (V+VI+VII) x Meritocracy (BE)				
Intermediate class (III+IV) x Merit: Effort (BE)			0.310^{**}	
			(0.119)	
Service class (I+II) x Merit: Effort (BE)			0.238^{*}	
			(0.098)	
Retired or pensioner x Merit: Effort (BE)			0.028	
			(0.108)	
Unemployed x Merit: Effort (BE)			0.040	
			(0.146)	
Performs unpaid tasks x Merit: Effort (BE)			−0.097	
			(0.108)	
Intermediate class (III+IV) x Merit: Talent (BE)				0.306^{**}
				(0.109)
Service class (I+II) x Merit: Talent (BE)				0.213^{*}
				(0.090)
Retired or pensioner x Merit: Talent (BE)				−0.070
				(0.100)
Unemployed x Merit: Talent (BE)				−0.026
				(0.137)
Performs unpaid tasks x Merit: Talent (BE)				−0.196
				(0.103)
Controls	Yes	Yes	Yes	Yes
BIC	40144.906	40135.120	40135.747	40100.765
Numb. obs.	15724	15724	15724	15724
Num. groups: individuals	3784	3784	3784	3784
Var: individuals (Intercept)	1.042	1.080	2.485	0.862
Var: individuals, wave	0.009	0.012	0.009	
Var: individuals, merit effort cwc	0.123			
Var: individuals, merit talent cwc		0.120		
Var: individuals, merit effort mean			0.321	

Note: Cells contain regression coefficients with standard errors in parentheses. $^{***}p < 0.001$; $^{**}p < 0.01$; $^{*}p < 0.05$. CWC = centered within group.
